# Comment on Pang et al. Ablative Techniques for Lung Metastases: Patient Selection and Outcomes Following Treatment with Stereotactic Radiotherapy or Radiofrequency Ablation. *Curr. Oncol.* 2025, *32*, 303

**DOI:** 10.3390/curroncol32090517

**Published:** 2025-09-17

**Authors:** Fergus Macbeth, Tom Treasure

**Affiliations:** 1 Centre for Trials Research, Cardiff University, Cardiff CF14 4YS, UK; 2 Clinical Operational Research Unit, Department of Mathematics, University College London, London WC1H 0BT, UK

We were interested to read the article by Pang et al. describing a retrospective review of 176 patients with lung metastases from a variety of primary tumours who were treated with either RFA or SABR at the Royal Marsden Hospital over 8 years [1]. It clearly showed how the different modalities were used for different tumours and suggested that SABR might in some circumstances be less effective than RFA at providing even local control. It also documents not insignificant adverse sequelae from both SBRT and RFA.

However, this study did not, nor could not, address the fundamental question as to whether removing or ablating lung metastases is actually effective in improving the patients’ most important clinical outcomes—overall survival (OS) and quality of life. In the Introduction Section they state that there is ‘a growing body of evidence for its survival benefit’. We have recently published a review in BMJ that shows, despite enthusiastic advocates worldwide, how insubstantial this evidence is [2].

In support of the view that SBRT might improve overall survival in patients with lung metastases they cite publications describing the long-term outcomes of two Phase II randomised trials [3,4] (Palma et al., Gomez et al.). Both were small studies including only 99 and 49 patients respectively. As we have pointed out before, in the Palma trial the two arms were imbalanced in favour of the intervention arm, which included patients with lower-stage and better-prognosis tumours [5]. The hazard ratio for OS was 0.57 but with very wide 95% confidence intervals. The Gomez et al. trial included only patients with non-small cell lung cancer. The primary outcome was progression-free survival, an unreliable outcome in ablation trials, as the identified sites of metastatic disease are those most likely to progress in the untreated arm. The trial was stopped early, and in the review of OS, only 28 patients were at risk at 2 years and 8 at 4 years—tiny numbers. Although there appeared to be a survival difference favouring intervention, it was not statistically significant and highly liable to chance variation. Overall, this is very poor evidence of effectiveness to justify the widespread uptake of this intervention. To our knowledge there is no good evidence supporting the use of RFA in this situation.

They cite the PulMiCC trial, of which we were co-investigators, but they only cite the initial publication. Further publications analysing a larger group of randomised patients [6] and the observational registration study in which the RCT was nested [7] lead us to believe that the major determinant of OS was patient selection rather than the intervention. Even with the small numbers randomised we have shown that a significant survival benefit is very unlikely (Figure 1).

It is time that the ‘routine’ use of SBRT and RFA for the treatment of asymptomatic metastases was paused, allowing reliable randomised trial evidence of clinical effectiveness to be generated [2].

## Figures and Tables

**Figure 1:**
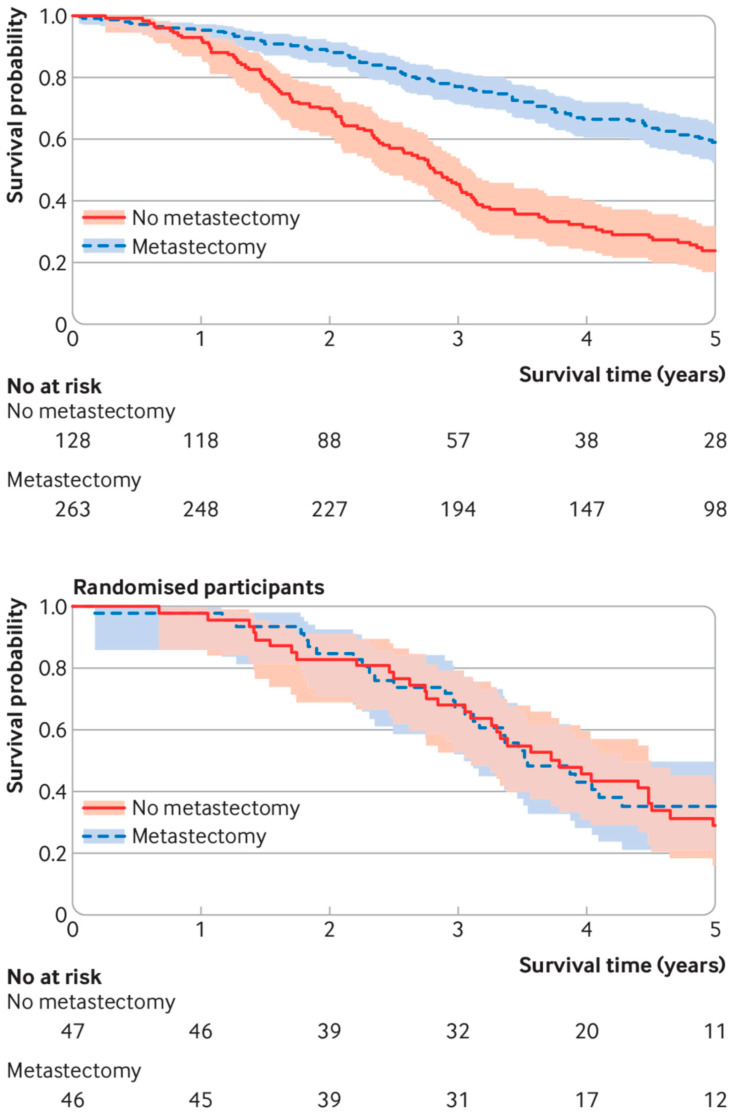
Survival curves for patients in the PulMiCC study. Of 484 patients with colorectal lung metastases with baseline and follow-up data collected to trial standards, 263 were selected by the clinical teams for metastasectomy and 128 were selected to NOT have metastasectomy (upper panel). Those selected for surgery had fewer metastases; the majority did not have raised carcinoembryonic antigen; and they had a better cancer stage, less liver involvement, better performance status, better lung function, and were younger. Their survival results were similar to many observational studies. In the nested controlled trial (lower panel), there was good balance for all known factors in the randomly assigned arms. There was no hint of a difference in survival.
